# Antitumor effects of acupuncture and moxibustion: from immune modulation to tumor microenvironment remodeling

**DOI:** 10.3389/fimmu.2026.1772199

**Published:** 2026-06-18

**Authors:** Yinan Li, Xia Gao, Tian He, Benxu Ma, Yonghui Zhao

**Affiliations:** 1 Department of Pathology, Qingdao Traditional Chinese Medicine Hospital, Qingdao Hiser Hospital Affiliated of Qingdao University, Qingdao, China; 2 Department of Acupuncture and Moxibustion, Qingdao Central Hospital, University of Health and Rehabilitation Sciences, (Qingdao Central Hospital), Qingdao, China

**Keywords:** acupuncture, antitumor effect, immunologic mechanism, moxibustion, tumor immune microenvironment

## Abstract

Acupuncture and moxibustion are evidence - based modalities derived from traditional Chinese medicine. They employ strategic needle insertion or moxa - derived thermal stimulation at specific acupoints to ameliorate pathological conditions. Robust clinical data have validated their efficacy in mitigating gastrointestinal dysfunction, chemotherapy - induced neuropathy, cancer - related fatigue, and radiation - induced xerostomia. Crucially, within immunosuppressive tumor microenvironments (TME), these interventions exert direct antitumor effects through multimodal immunomodulation (1): Potentiation of innate immunity via enhanced cytotoxicity of *natural killer (NK) cells*, polarization of *macrophages* toward the proinflammatory M1 phenotype, regulation of microglial function, and modulation of *mast cell (MC)* degranulation; (2) Reprogramming of adaptive immunity through restoration of the *T helper 1 (Th1)/T helper 2 (Th2)* balance and activation of cluster of differentiation 8 - positive *(CD8^+^) T cells*; (3) Remodeling of the TME by attenuating immunosuppressive networks (e.g., *regulatory T cells ((Tregs)*, myeloid - derived suppressor cells ((*MDSCs*)) while enhancing effector cell infiltration. This review synthesizes mechanistic advances that demonstrate how acupuncture and moxibustion rebalance antitumor immunosurveillance, thereby positioning them as promising adjuvant modalities in integrative oncology.

## 1 Introduction

Tumor development is closely linked to the immune system of each individual (1, 2). Immunotherapy has emerged as a transformative clinical strategy for cancer treatment (3), representing a paradigm shift from conventional cytotoxic approaches by specifically harnessing host immune mechanisms to combat malignancies. This therapeutic modality aims to enhance the patient’s immunity to effectively target and eliminate cancer cells (4),​ primarily through the activation or augmentation of immune responses that recognize and destroy tumor cells via natural defense pathways frequently evaded during disease progression.

Acupuncture, a cornerstone of traditional Chinese medicine, has been shown to modulate the host immune function and regulate pathological conditions, offering effective relief or treatment for associated diseases (5, 6). This ancient diagnostic and therapeutic technique has been widely practiced in China for thousands of years. Acupuncture encompasses a variety of techniques, including manual acupuncture (MA), electroacupuncture (EA) therapy, catgut implantation at acupoints (CIAA) therapy, and grain-sized moxibustion (gMoxi) therapy. In traditional Chinese medicine (TCM), cancer arises from deficiency of Zhengqi (healthy qi) and excess of Xieqi (pathogenic factors). The therapeutic principle of Fuzheng (supporting Zhengqi) corresponds to enhancing immune surveillance and defensive functions, while Quxie (eliminating Xieqi) mirrors the elimination of tumor cells, thus rebalancing the immune system to combat cancer (7). Additionally, the associated hypothesis between the theory of qi and blood and TME metabolic regulation posits that “blood stasis” induced by qi deficiency can cause local ischemia and hypoxia in the TME. This condition triggers abnormal metabolic patterns in tumor cells and imbalances in angiogenesis, creating a vicious cycle of “blood stasis-hypoxia-tumor progression”. Numerous studies have demonstrated that acupuncture can regulate the body’s immune functions, improve the tumor immune microenvironment (TIME), and thereby exert potential antitumor effects (8, 9).

This manuscript aims to review the immunomodulatory effects of acupuncture therapy in the context of tumor immunity. It will cover the regulation of innate immune cells such as *NK cells*, *macrophages*, *microglia*, and *MCs*, as well as adaptive immune cells including cluster of differentiation 4-positive (*CD4^+^)T cells* and *CD8^+^T cells*. Additionally, it will explore how acupuncture influences the secretion of immune-related cytokines, nervous pathways and modulates immune responses within the TME, thereby contributing to exploring the research progress of the antitumor mechanism of acupuncture.

## 2 Modulation for non-specific immune cells

### 2.1 Natural killer cells

*NK cells* are a key component of innate immunity and play a central role in host defense against cancer and pathogens (10–12). Previous studies have demonstrated that acupuncture upregulates the functions of *NK cells* to enhance antitumor immunity. Acupuncture is closely related to anti-cancer effects by regulating *NK cells*, and this process is closely associated with the cytokines β-endorphin (β-EP), interferon-γ (IFN-γ), and IL-2 (13).

Acupuncture, especially when stimulating ST36 (Zusanli), induces nitric oxide synthase (NOS) in keratinocytes, leading to increased nitric oxide (NO) concentration increased. NO transmits signals to neural centers in the brain, promoting the production of β-EP. The β-EP binds to opioid receptors on *NK cells* membranes, activating *NK cells* and stimulating the production of IFN-γ. IFN-γ subsequently promotes IL-2 secretion by other immune cells, which strongly stimulates *NK cells* and enhances their cytotoxic activity for direct cancer cells killing. This mechanism not only amplifies the innate immune response but also primes the tumor microenvironment for synergistic interactions with immunotherapies (14).

Acupuncture can significantly enhance *NK cells* activity by increasing the secretion of IFN-γ, with clinical timing including perioperative administration (e.g., 15 min before anesthesia until surgery completion) or during chemotherapy (e.g., once daily, 5 days per week), commonly using acupoints like ST36 and SP6 (Sanyinjiao) for 15–30 minutes per session (15). IFN-γ regulates *NK cells* activity by increasing the expression of tumor necrosis factor (TNF) superfamily ligands and *NK cells* receptors and stimulating cytokine secretion through other immune cells. Furthermore, IL-2 not only stimulates *NK cells* but also promotes the expression of the NKp44 receptor on *NK cells* surfaces, activating *NK cells* cytotoxicity (14).

In colorectal cancer (CRC) patients, acupuncture promotes *NK cells* proliferation and activity, which stimulates the hypothalamic-pituitary-adrenal (HPA) axis to secrete β-EP. β-EP binds to *NK cells* surface receptors, promoting cytotoxic molecule expression and IFN-γ production to enhance antitumor immunity, while alleviating depression and anxiety levels. Clinically, acupuncture is initiated one week before chemotherapy, administered twice weekly for 6 sessions (45 min/session), and terminated at the start of the next chemotherapy cycle. The standardized protocol involves needling lower extremity acupoints (LV3 (Taichong), ST36, SP3 (Taibai), GB39 (Xuanzhong)) and upper extremity acupoints (LI4 (Hegu), PC5 (Jianshi), TB5 (Waiguan), LU7 (Lieque)) with 36G × 25 mm needles to a depth of 10 mm to elicit deqi, combined with smokeless moxibustion (2 min/acupoint) at SI6 (Yanglao), TB5, ST32 (Futu), and CV6 (Qihai). This regimen significantly increases *NK cells* counts (2-fold increase at T3, P = 0.000), white blood cell (WBC, P = 0.036), and absolute neutrophil counts (ANC, P = 0.046), with immunomodulatory effects correlated to needling intensity (deqi sensation) and moxibustion duration (2 min/acupoint). Although the acupuncture protocol did not produce statistically significant between-group differences in overall quality of life scores, it demonstrated measurable benefits in specific areas-particularly in mitigating chemotherapy side effects and improving psychological well-being-highlighting its potential as a valuable supportive therapy for patients undergoing chemotherapy (16).

EA at ST36 was administered 1 day after each cisplatin chemotherapy cycle (50 mg/m²/week) for 4 cycles, with 0.25×40 mm needles inserted to 10–15 mm depth, with 2 Hz frequency, 0.2 mA intensity, and 30 min duration. This intervention increased the proportion of peripheral blood *NK cells* and reduced tumor volume in patients with stage IIb - IIIb cervical squamous cell carcinoma (SCC). Researchers proposed that enhanced *NK cells* activity was mediated by EA-induced IFN-γ upregulation, although dose-response data for parameters such as intensity remain unreported (17).

EA at ST36 modulates interleukin-1β (IL-1β) and tumor necrosis factor-α (TNF-α) to reduce inflammation, thereby inhibiting breast cancer growth in tumor-bearing mice. Specifically, EA intervention was initiated when tumor volume reached ≥50 mm^3^ (10 days postimplantation of 4T1-luc2 cells), applied bilaterally at ST36 with 0.1 mA intensity and 2/15 Hz frequency for 30 min every other day, lasting until day 22. Furthermore, EA intervention significantly increased perforin and granzyme B (GzmB) expression in local tumor tissue, indicating augmented cytolytic activities of *NK cells* and *CD8^+^ T cells* to enhance antitumor immunity, which was mediated by activation of efferent vagus nerve activity (18). In the 4T1 murine breast cancer model, sciatic nerve stimulation robustly activates the D1-like dopamine receptor-cAMP-PKA-cAMP response element-binding protein (CREB) signaling cascade, which is crucial for enhancing the cytotoxicity of *NK cells* and suppressing tumor progression. Concurrently, neural stimulation increases serum IFN-γ, upregulates programmed death-ligand 1 (PD-L1) expression, and thus sensitizes breast cancer cells to immunotherapy (19).

In a Lewis lung cancer (LLC) xenograft model, grain-sized moxibustion (gMoxi) at ST36 significantly inhibited tumor growth by promoting *NK cells* antitumor immunity through adrenergic signaling inhibition. Administered from day 1 post-tumor inoculation, gMoxi used 3 or 7 moxa cones at ST36 every other day-a regimen showing superior efficacy over daily or weekly schedules and suggesting early clinical initiation alongside conventional non-small cell lung cancer (NSCLC) therapies. Notably, 3 and 7 moxa cones yielded comparable increases in *NK cells* proportion, tumor infiltration, and activation, indicating a non-linear dose-response. The mechanistic analysis showed that gMoxi could promote *NK cells* antitumor immunity by inhibiting adrenergic signaling, resulting in tumor regression. Specifically, gMoxi enhanced *NK cells*-mediated cytotoxicity. Since *NK cells* can eliminate target cells without prior sensitization, the enhanced splenocyte-mediated cytotoxicity after gMoxi treatment is primarily attributed to *NK cells* activation. These findings further confirm that gMoxi effectively enhances the capacity of *NK cells* to eliminate tumor cells, offering a novel perspective and potential therapeutic strategy for NSCLC treatment (20).

**Figure 1:**
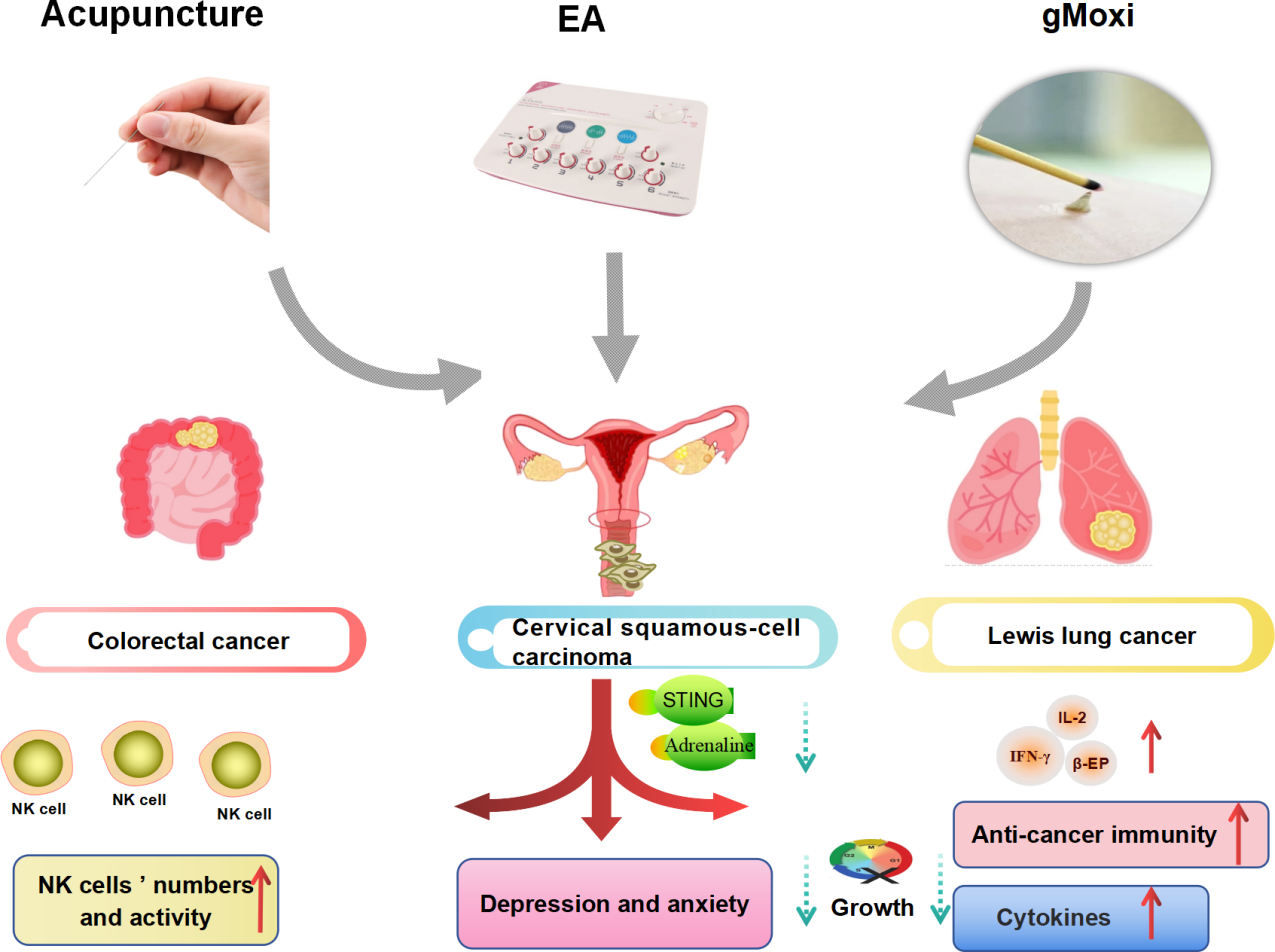
Regulation of NK cells by acupuncture therapies and their impacts on anti-cancer immunity and neuroendocrine-immune networks. Intervention factors (acupuncture, EA, gMoxi) activate the STING pathway while inhibiting adrenergic signaling. This mechanism enhances the release of β-EP, IFN-γ, and IL-2, thereby increasing peripheral blood NK cells proportion, upregulating NK cells activity, reducing tumor volume in cervical squamous-cell carcinoma, and suppressing Lewis lung cancer growth. Furthermore, this immunotherapeutic approach not only enhances anti-tumor immunity in colorectal cancer patients but also ameliorates symptoms of depression and anxiety symptom. EA, electroacupuncture; gMoxi,grain-sized moxibustion; NK, natural killer; IFN-γ, interferon-gamma; IL, interleukin; STING, stimulator of interferon genes; β-EP, β-endorphin.

Anti-programmed death-1(anti-PD-1) therapy relieves tumor-mediated immunosuppression of T cells, effectively treating microsatellite instability (MSI)-type CRC, but combination strategies are needed for microsatellite stability (MSS)-type CRC. Wang et al. first demonstrated that EA synergizes with anti-PD-1 to trigger robust antitumor immunity in MSS-CRC by remodeling the immunosuppressive TME. Mechanistically, EA upregulates intratumoral interferon-β (IFN-β) and GzmB, enhances infiltration of cytotoxic immune cells (GzmB^+^ *NK/NKT cells* and *CD8^+^ T cells*), and activates the stimulator of interferon genes (STING) pathway to amplify antitumor responses. The protocol involves bilateral ST36 acupoint stimulation with EA at 10 Hz, 1.0 mA intensity for 30 min daily for 14 consecutive days. Dose-response data revealed that 1.0 mA is optimal, outperforming 0.5 mA and 1.5 mA in tumor inhibition by modulating immune cells infiltration and STING pathway activation, establishing a translational framework for combining EA with anti-PD-1 in MSS-CRC (21) (**Figure 1**; **Table 1**).

**Table 1:** The effects of acupuncture and moxibustion therapy in regulating anti-cancer. ↑ increased / upregulated; ↓ decreased / downregulated.

Therapy	Acupoints and dosage	Immune efficacy	Mechanism/pathway	Cancer type	Reference
Acupuncture and Smokeless moxibustion	6 sessions,biw; Disposable ower extremity (LV3, ST36, SP3, GB39) upper extremity (LI4,PC5, TB5, LU7). Size:36-G,0.20-x-25-mm (Tewa), l0.mm. Smokeless moxibustion SI6, TB5, ST32, CV6 2 min/point, 45min	NK cells 2x increase; WBC 1.5x reduction in leukopenia rates; ANC 1.5x reduction in neutropenia rates.	-	CRC	(16)
EA	ST36: 1d after chemotherapy (4 cycles); 2 Hz, 0.2 mA, 30 min	NK cells (%) (-5.0731(4.96) vs. 0.8219 (3.30), P = 0.00)	-	SCC	(17)
EA	ST36 0.1 mA, 2/15Hz, 30min, QOD	↓IL-1β, TNF-α (P < 0.01 in serm, P < 0.01 in tumor); ↑Perforin (P < 0.05), granzyme B (P < 0.01); ↑*CD8*^+^ T cells, NK cells (proportion, cytolytic activities) ↓MDSCs (accumulation levels, immunosuppressive capacity)	subdiaphragmatic vagus nerve activation (c-Fos↑)	BC	(18)
Grain-sized moxibustion (gMoxi)	ST36 (3 moxa cones, QOD)	↑ NK cells (proportion, Infiltration, activation in tumor P < 0.05 and spleen P < 0.001)	adrenergic signaling inhibiton (↑Cacna1s, Cacng1, Myl2, Myh7, Myh6, Myl3, Atp1a2, Scn4b, Tnnc1and Actc1; ↓Tnnt2)	LLC	(20)
EA	ST36 (10 Hz,1.0 mA, 30 min/d for 2weeks)	↑IFN-β (P<0.01), GzmB^+^NK cells(P<0.01), GzmB^+^NKT cells (P<0.01), *CD8*^+^T cells (P<0.05); ↓MDSCs(P<0.05)	STING signaling pathway activation (pTBK1 and pIRF3, P < 0.01)	CRC	(21)

Acupuncture does not merely “enhance” *NK cells* function in the general sense. It participates in the neuro-immune axis (ST36 → NO → β-endorphin → opioid receptors → NK activation) and promotes the production of IFN-γ and IL-2. Moreover, ST36, as a core acupoint, can induce qualitatively distinct remodeling of the TME depending on stimulation modalities (manual acupuncture, electroacupuncture, moxibustion) and parameters. EA at 1.0 mA/10 Hz achieved STING-dependent TME remodeling in MSS-CRC, indicating that the immune threshold relies on specific parameters rather than a linear dose-response relationship. Acupuncture reduces the accumulation of MDSCs and tumor-associated macrophages (TAMs), increases *CD8^+^ T cell/NK cell* co-infiltration, relieves neuroendocrine suppression, and improves psychological status-the latter may directly attenuate the inhibitory effects of HPA-driven glucocorticoids on *NK cells*. Collectively, acupuncture regulates *NK cells* function through multiple mechanisms, including increasing *NK cells* number, activity, and cytotoxicity, promoting *NK cells* proliferation and differentiation, remodeling the immune microenvironment, activating immune signaling pathways, and directly inducing tumor cell death.

### 2.2 Macrophages

*Macrophages* are essential in innate immunity. Activated m*acrophages* can be sub-categorized into *M1* (proinflammatory) or *M2 macrophage(M2)* (anti-inflammatory) phenotypes (22, 23). Recent studies have demonstrated that acupuncture plays a pivotal role in the immunomodulation of tumors and inflammatory diseases by regulating the polarization and functions of *macrophages*.

Acupuncture has been observed to activate *macrophages* and enhance their tumoricidal activity against tumor cells. *Macrophages* stimulated by acupuncture produce increased levels of nitric oxide (NO), TNF, and interleukins, which contribute to tumor cell destruction. *Macrophages* recruited to the tumor area are polarized into the *M1* type that inhibits tumor growth or the *M2* type that promotes tumor growth (24, 25). Tumor-associated *microglia*/*macrophages* (TAMs) predominantly play an *M2*-like tumor-promoting role in TME (26). Notably, inducing the polarization of TAMs towards the *M1* phenotype offers multiple therapeutic benefits. It can normalize tumor blood vessels, thereby improving the delivery of anticancer agents. Additionally, this polarization significantly enhances antitumor immunity, *M1*-like TAMs inhibit tumor growth through multiple mechanisms: secreting proinflammatory cytokines (e.g., IL-12, IL-6, IFN-γ) to activate antitumor immunity, reducing *M2*-associated immunosuppressive cytokines (e.g., IL-10, transforming growth factor-β (TGF-β)) and immunesuppressive cells (e.g., *Treg*s, *MDSCs*), downregulating nuclear factor κB p50 (NF-κB p50) and Rho-associated kinase 2 (ROCK2) to block *M2* polarization and angiogenesis, and inducing tumor cell apoptosis via inducible NOS (iNOS)-mediated NO production. Engineered *M1* exosomes (IL4R-Exo(si/mi)) decorated with IL4RPep-1 peptide enhance tumor targeting of *M2* TAMs, efficiently delivering NF-κB p50 siRNA and miR-511-3p to reprogram *M2* TAMs into *M1*-like phenotypes, thereby IL-10 suppressing tumor progression through synergistic reprogramming of the tumor microenvironment and enhanced antitumor immune responses (27).

A recent study confirms for the first time that peritumoral EA promotes *M1* TAMs in the 4T1 breast cancer model. A 30 min EA was performed on the day when the tumor volume reached 100–300 mm^3^ in 4T1 breast cancer xenograft mice, using peritumoral multi-needling at 1–2 cm from the tumor edge with four needles angled 15° toward tumor (depth 15–20 mm for superficial tumors), applied with dense-sparse wave (sparse wave 3–4 Hz/5 s, dense wave 15–20 Hz/10 s) at 1–3 mA intensity, 3 sessions/week for 3 weeks. EA reshapes the TME by polarizing TAMs toward an *M1* phenotype through dual mechanisms: upregulating proinflammatory cytokines TNF-α and IL-1β while downregulating anti-inflammatory cytokines TGF-β and interleukin-10 (IL-10). Concurrently, EA inhibits angiogenic factors vascular endothelial growth factor (VEGF), placental growth facto (PIGF), matrix metalloproteinase 2 (MMP2), and MMP9, reducing microvascular density, promoting vascular maturation, and suppressing tumor growth, thus leading to normalization of tumor vasculature and enhanced antitumor immunity. Mechanistically, EA downregulates glyoxalase-1 (GLO1), triggering accumulation of methylglyoxal (MGO) and activation of the MGO-advanced glycation end products/receptor for advanced glycation end products (MGO-AGEs/RAGE) axis, which reprograms TAMs to an *M1* phenotype and disrupts proangiogenic signaling (28).

Moxibustion intervention, performed at bilateral ST36 acupoints for 15 min five times per week for two weeks, starting one week after tumor cell transplantation, was confirmed to promote the polarization of TAMs to the *M1* phenotype and enhance the expression of *M1 cells* surface molecule cluster of differentiation 86 (*CD86*) when combined with cisplatin in treating NSCLC. As a positive co-stimulatory molecule, *CD86* provides signals for *T cells* activation, thus enhancing the antitumor immune response. Immune-vascular combined application therapy exerts its efficacy through multiple mechanisms: increasing infiltration of immunostimulatory cells *(M1 macrophages*, *CD8^+^ CTLs*, *CD4^+^T cells*, *Th1*, *Th9* subsets), elevating interferon-gamma (IFN-γ) and IFN-g gene expression in tumors, promoting pericyte coverage to normalize tumor vasculature, and inhibiting angiogenesis via downregulation of vascular endothelial growth factor (VEGF). These effects improve vascular structure, reduce hypoxia, and promote cytotoxic T lymphocyte infiltration in the TME. Consequently, they enhance antitumor immunity and therapeutic efficacy (29).

Another study has found that acupuncture can also alleviate the side effects of chemotherapy drugs by regulating the polarization of macrophages. EA can alleviate signs of paclitaxel-induced peripheral neuropathy (PIPN) in model animals by reducing proinflammatory *macrophage* infiltration in peripheral sensory ganglia and nerves. The EA group received acupuncture at ST36 and BL60 (Kunlun) acupoints, connected to a HANS stimulator at 2 Hz, 0.2 ms pulse width, 0.5 mA intensity for 30 min, daily for 7 consecutive days starting 1 day after the 4th paclitaxel injection; the sham EA group underwent subcutaneous needle insertion without electric stimulation at the same acupoints and timing. Mechanistically, EA may affect C-C motif chemokine ligand 2 (CCL2) overexpression in dorsal root ganglion (DRG) neurons to reduce *macrophage* infiltration. Additionally, EA may also intervene in interleukin-33/suppression of tumorigenicity 2 (IL-33/ST2) signaling involved in *macrophages* infiltration and produce an anti-allodynic effect on PIPN model mice (30).

**Figure 2:**
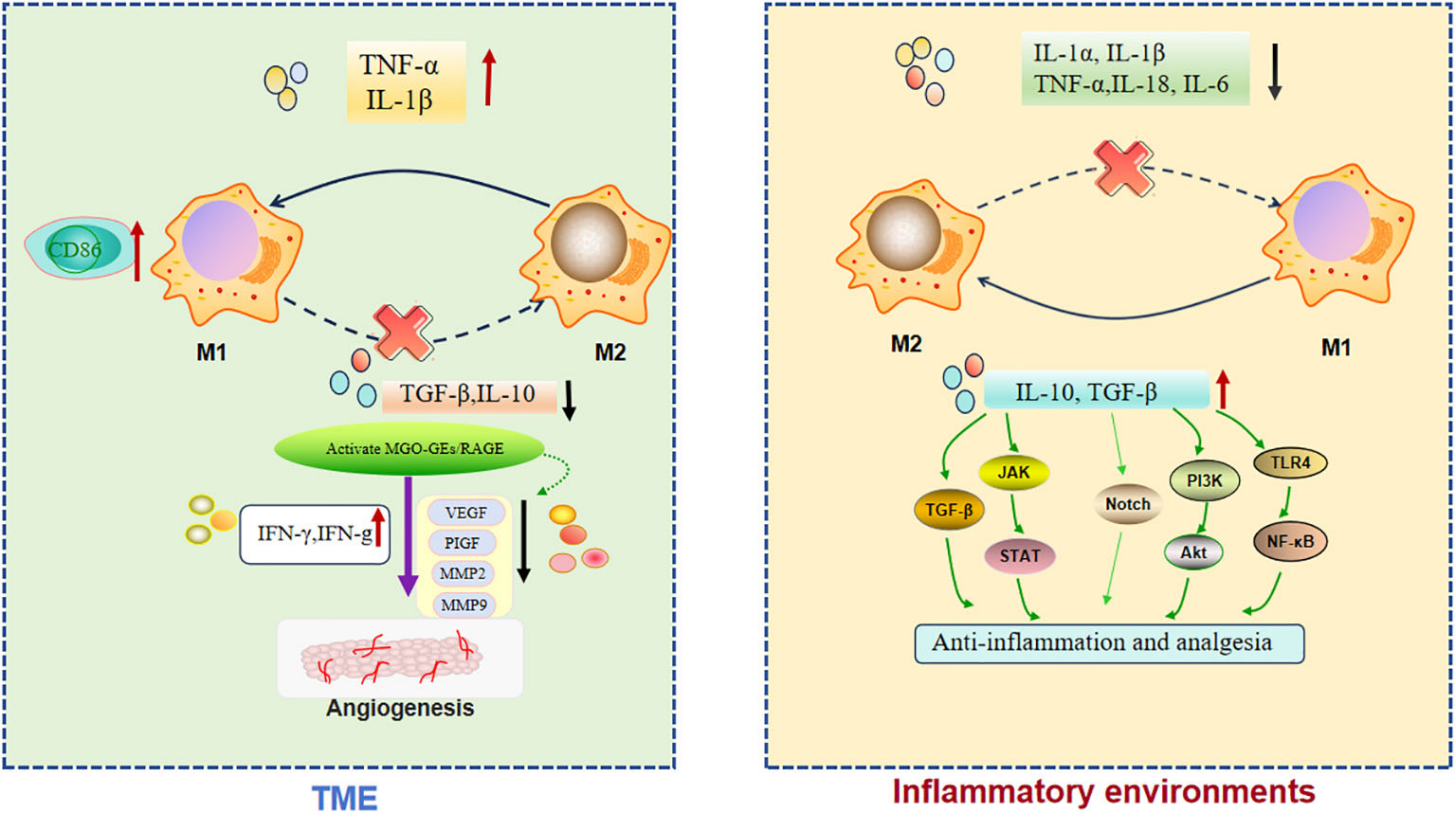
Phenotypic polarization of macrophages and their regulatory cytokines in TME and inflammatory environments. In TME, intervention factors promote M1 TAMs polarization by upregulating TNF-α/IL-1β, downregulating TGF-β/IL-10, and activating the MGO–AGEs/RAGE axis via GLO1 inhibition. This suppresses angiogenic factors (VEGF/PIGF/MMP2/MMP9), promotes vascular maturation, and elevates IFN-γ and IFN-g to normalize tumor vasculature and inhibit angiogenesis. In inflammatory environments (e.g., arthritis), intervention factors inhibit M1 polarization and reduce pro-inflammatory factors (IL-1α/β, TNF-α, IL-18, IL-6) in inflamed joints while increasing M2 macrophages and anti-inflammatory cytokines (IL-10, TGF-β). This process involves regulating signaling pathways (PI3K/Akt, Notch, JAK-STAT, TGF-β, and TLR4/NF-κB) to exert anti-inflammatory and analgesic effects. M1, pro-inflammatory macrophage; M2, anti-inflammatory macrophage; TME, tumor microenvironment; TNF-α, tumor necrosis factor-α; IL-1β, interleukin-1 beta; TGF-β, transforming growth factor-β; IL-10, interleukin-10; VEGF, vascular endothelial growth factor; PIGF, placental growth factor; MMP2, matrix metalloproteinase 2; Akt, protein kinase B; JAK-STAT, janus kinase-signal transducer and activator of transcription; NF-κB, nuclear factor-κB; TLR4, toll-like receptor 4.

In addition, in the process of chronic inflammatory diseases treatment, acupuncture alleviates inflammatory responses and ameliorates clinical symptoms by regulating the phagocytic function of *macrophages* (31, 32). MA at ST36 can predominantly attenuate the *M1-*like *macrophages* polarization by upregulating TGF-β1, IL-10 expression, and downregulating related factors (IL-1α, IL-1β, TNF-α, IL-18, IL-6) expression, thus achieving anti-inflammatory and analgesic effects within the inflammatory immune microenvironment (33). These findings demonstrate that acupuncture promotes the transformation of *macrophages* in inflammatory disease from the *M1* to the *M2* phenotype. In addition, acupuncture has been proven to regulate the following signaling pathways and transcription factors to polarize *macrophages*: phosphoinositide 3-kinase/protein kinase B (PI3K/Akt) signaling pathway, the Notch signaling pathway, the Janus kinase-signal transducers and activators of transcription (JAK/STAT) signaling pathway, the TGF-β signaling pathway, and the toll-like receptor 4/NF-κB (TLR4/NF-κB) signaling pathway (34) (**Figure 2**).

In summary, the regulatory effect of acupuncture on *macrophages* is not confined to simple “activation” or “inhibition”. Instead, acupuncture exerts a bidirectional regulatory role depending on the specific microenvironment: in tumor tissues, it induces the polarization of TAMs toward the *M1* phenotype, whereas in inflammatory disorders, it promotes the polarization of *M1* macrophages to the *M2* phenotype. Notably, both regulatory processes are involved in the modulation of the NF-κB signaling pathway, suggesting that acupuncture assists the immune system in restoring homeostasis rather than exerting a fixed directional effect. In addition, peritumoral EA contributes to the normalization of the tumor vascular system, characterized by reduced expression of VEGF and MMP2/9, as well as increased pericyte coverage, while concurrently repolarizing TAMs toward the *M1* phenotype. This dual regulatory effect-combining immune modulation and vascular normalization-may underlie the systemic antitumor efficacy of local acupuncture intervention. Furthermore, as an adjuvant therapeutic strategy, acupuncture can alleviate chemotherapy-induced side effects such as peripheral neurotoxicity (PIPN), enhance the therapeutic efficacy of chemotherapeutic agents including cisplatin and paclitaxel, and mitigate chemotherapy resistance. Collectively, these findings provide a novel perspective for the development of adjuvant treatment strategies in the field of oncology.

### 2.3 Microglia

*Microglia* are the primary innate immune cells within the central nervous system, which are phagocytes present in the brain parenchyma (35, 36). In the TME, *microglia* have the ability to polarize to either a proinflammatory phenotype *(M1*), or an anti-inflammatory phenotype (*M2*). The *M2* phenotype is immunosuppressive and promotes tumor growth by secreting growth factors or facilitating angiogenesis, while the *M1* phenotype is characterized by the secretion of proinflammatory cytokines and enhances antitumor immune responses. In mouse models and human patients with brain metastases (BrMs), *microglia* are predominantly of the *M2* phenotype. Upregulation of heat shock protein 47 (HSP47) in metastatic tumor cells enhances collagen type 1 (COL1) biosynthesis and deposition in metastatic lesions, thereby driving *M2 microglia*l polarization (36–38).

*Microglia* regulate tumor progression through cytokine/chemokine signaling (e.g., IL-6, C-X-C motif chemokine ligand 1 (CXCL1)), immune cell polarization, and matrix remodeling, with context-dependent roles influenced by tumor type and TME (39). In glioblastoma (GBM), the glioma microenvironment exhibits specificity wherein isocitrate dehydrogenase (IDH) mutation status impacts therapeutic efficacy by regulating immune cell infiltration, transcriptional profiles of glioma-associated *microglia*/*macrophages*, and immunosuppressive signaling pathways, with IDH wild-type tumors maintaining a robust immunosuppressive niche resistant to immunotherapy and IDH-mutated tumors potentially responding to interventions targeting metabolic dysregulation or *microglial* phenotypic reprogramming. On the other hand, *microglia* can exert antitumor effects: enriched environmental stimuli enhance *microglial* interleukin−15 (IL-15) secretion, activating *NK cells* to suppress tumor growth (40, 41).

Additionally, EA may relieve chemotherapy-induced peripheral neuropathy (CIPN) by inhibiting *M1 microglia* and proinflammatory cytokines, such as IL-1β, IL-6, and TNF-α, in the spinal cord of cisplatin-treated mice and increase the expression of anti-inflammatory cytokines (such as IL-4 and IL-10), which are consistent with the mechanism by which scalp acupuncture (SA) in middle cerebral artery occlusion (MCAO) rats modulates the activity of *microglia* to reduce nerve damage in the treatment of ischemic stroke. Further research confirms that EA promotes the release of miR-124 in the spinal cord of mice treated with cisplatin, and regulates microglial activity and neuroinflammation, thereby alleviating CIPN (42).

### 2.4 Mast cells

*Mast cells* (*MCs*) are crucial cells participating in both innate and adaptive immune processes, playing both tumor-promoting and tumor-suppressing roles depending on the context (43, 44). When exposed to a TME rich in hypoxia, acidity, stem cell factor (SCF) and chemokines such as CCL2, MCs often release protumorigenic mediators. However, under different conditions-such as in immune-stimulatory TMEs or upon proinflammatory signal activation-*MCs* can shift to an antitumor role by releasing cytokines (e.g., IL-1, IL-4, IL-6, TNF-α) that promote tumor cell elimination and rejection. Conversely, MC-derived factors such as fibroblast growth factor-2 (FGF-2), nerve growth factor (NGF), platelet-derived growth factor (PDGF), VEGF, IL-8, and IL-10 have been shown to promote tumor cell proliferation (45).

Acupuncture stimulation can activate and induce degranulation of peripheral *MCs*, which play a significant role in pain relief and immune modulation. Acupuncture can deform collagen fibers and activate transient receptor potential vanilloid 1 (TRPV1) channels on *MCs* membranes, thereby stimulating *MCs* to release bioactive substances (such as adenosine triphosphate (ATP), substance P (SP), tryptase, histamine (HA), interleukins, 5-hydroxytryptamine (5-HT)). Acupuncture can improve neuropathic pain and hot flashes in cancer survivors by activating the *MC* of acupoints via TRPV1 and adenosine A1 receptor (ADORA1) pathways (46).

Although current research on the direct effects of acupuncture on tumors via *MCs* regulation remains limited, acupuncture shows potential in modulating *MC*-mediated immune responses within the TME. By activating *MCs* to secrete cytotoxic cytokines (e.g., TNF-α, IL-1), acupuncture may enhance tumor cell elimination and inhibit tumor-promoting pathways. Future studies are warranted to investigate how acupuncture orchestrates *MC*-mediated crosstalk within the TME, such as enhancing antitumor immune cell infiltration (e.g., *CD8^+^ T cells*) or regulating proinflammatory/anti-inflammatory cytokine balance, to clarify its role in synergizing with conventional cancer treatments.

### 2.5 Neutrophils

*Neutrophils* are the most abundant innate immune cells in peripheral blood and bone marrow. In cancer, neutrophils play dual roles as either pro-tumor (N2) or antitumor (N1) phenotypes (47). Mild moxibustion, administered once daily for 15 minutes at CV8 (Shenque), bilateral ST36, and bilateral Sanyinjiao (SP6) has been shown to modulate neutrophil function. In breast cancer patients, this intervention elevates white blood cell (WBC) and absolute neutrophil counts (ANC) throughout chemotherapy, with prominent effects observed in the 7th cycle-effects. It reduces the risk of grade 3–4 myelosuppression and febrile neutropenia, enhances chemotherapy compliance, and minimizes treatment interruptions. The mechanism may involve boosting granulocyte colony-stimulating factor (G-CSF) levels and modulating the neuroendocrine-immune network to support hematopoietic function, thereby indirectly curbing tumor progression by preserving antitumor immune surveillance (48).

Acupuncture modulates *neutrophils* to alleviate chemotherapy-induced myelosuppression, increasing neutrophil counts, absolute *neutrophil* counts, and improving immune function (49–51).

### 2.6 Dendritic cells

*Dendritic cells* (*DCs*) are key antigen-presenting cells (APCs) bridging innate and adaptive immunity. Tumor-associated *DCs* are primarily responsible for presenting antigens to effector *T cells* and producing cytokines to induce the antitumor immune response of *killer T cells*, especially *CD4^+^* and *CD8^+^ T cells* (52). In tumor tissues, *DCs* can initiate antitumor *T cells* response by processing and presenting tumor-associated antigens (TAAs), but the TME often impairs their recruitment and function through metabolites and cytokines, enabling immune evasion.

EA at CV 17 (Tanzhong) and ST36 potentiates the efficacy of anti-PD-1 therapy in breast cancer models through multi-dimensional immunomodulation. Transcriptomic analysis reveals that differentially expressed genes (DEGs) in tumors are enriched in immune-related pathways, including T cell receptor signaling, PD-1 checkpoint pathway, and *T cells* differentiation/activation. Key DEGs, such as cluster of differentiation 5 (CD5), C*D4, CD8, CD247*, and *CD28*, form an interaction network central to *T cells*-mediated immunity. Specifically, this acupoint combination promotes the intratumoral accumulation of *CD5^+^ DCs*-a DC subset critical for sustaining responsiveness to anti-PD-1 therapy by driving proinflammatory *T cells* polarization, while concomitantly expanding *CD4^+^* and *CD8^+^ T cells* populations and elevating the levels of proinflammatory cytokines (IL-2, IL-6, TNF-α, IFN-γ), thus potentiating tumor-specific cytotoxic immune responses (53). The *DC-T cell* axis is an effective target for the treatment of NSCLC (54), grain-sized moxibustion at ST36 in Lewis Lung Carcinoma (LLC) tumor models inhibits β-adrenergic signaling, promoting *DCs* maturation (upregulating major histocompatibility complex class II (MHC-II) and *CD86*), synergizing with cancer vaccines to activate *CD4^+^ T cells* and *NK cells*, thereby suppressing tumor growth (55). (**Table 2**).

**Table 2:** The effects of acupuncture and moxibustion therapy in anti-cancer immune or RA by regulating macrophages, microglia, neutrophils and dendritic cells. ↑ increased / upregulated; ↓ decreased / downregulated.

Cell type	Therapy	Acupoints and dosage	Immune efficacy	Mechanism/pathway	Cancer type	Reference
Macrophages	EA	Four points were inserted at the top, bottom, left, and right at a distance of 5 mm from the tumor edge. (3–4 Hz for 5 s and 15–20 Hz for 10 s, 30 min, tiw, for 3weeks)	↑M1 (M1/M2 ratio P<0.01; iNOS↑/Arg1↓ P<0.01); ↑TNF-α, IL-1β (P<0.01); ↓TGF-β, IL-10 (P<0.05)	Upregulate GLO1-MGO-AGE/RAGE pathway	BC	(28)
	Moxibustion	ST36 (15 min, 5 times/week, 2weeks)	↑M1, IFN-γ (P<0.01) ↓VEGF (P<0.01)	Improve vascularnormalization, suppress VEGF expression	NSCLC	(29)
	EA	ST36, BL60 (2 Hz, 0.5 mA, 30/d,7 days)	↓CCL2 (P<0.01)	Reduce proinflammatory Macrophage infiltration pathway mediated by CCL2/CCR2	PIPN	(30)
Microglia	EA	ST36, BL60 (100 Hz/2 Hz, 3s each), 1–2 mA current, 15 min/day, 7 days	↓IL-1β, TNF-α (P<0.01) ↑IL-10, IL-4 (P<0.05) ↓M1 (CD16/32, P<0.01); ↑M2 (CD206, IL-10)	Inhibite TREM2/DAP12	Chemotherapy-induced peripheral neuropathy (CIPN)	(42)
Neutrophils	Mild moxibustion	CV8, ST36, SP6 (15 min, moxa stick (1.5cm long) 21 days/cycle, 4–8 cycles total	↑ ANC, WBC (WBC: 5.38 ± 1.41 vs 3.78 ± 1.46×10^9^/L, P < 0.01; grade 1 ANC reduction: 0% vs 31.8%, P < 0.01)	-	BC	(48)
	Acupuncture	lower extremity (LR3, K3, SP6, ST36, SP10); upper extremity (LI4, PC6, LI11); and the top of head (GV20); EA(ST36 and SP6), 20–25 Hz, 30 min	-	-	Ovarian cancer	(49)
Dendritic cells	EA	CV17, ST36 (2 Hz frequency, 0.1–0.3 mA, 30 min)	↑DCs, *CD4*^+^ and *CD8*^+^ T cells (P<0.01) ↑IL-2 (P<0.01), IL-6 (P<0.05), TNF-α (P<0.01)	Activate PI3K/Akt and other signals	BC	(54)
	Grain-sized moxibustion	ST36 (cone-shaped moxa, 3 cones, qod)	↑DCs, *CD4*^+^ T cells and NK cells (P<0.05) ↓β-adrenergic	Inhibit β-adrenergic signaling	LLC	(55)

In summary, acupuncture has the potential to reshape the tumor microenvironment by modulating the function, polarization, and secretion profiles of *DCs*. The most extensively investigated mechanisms involve the activation of *NK cells* via the β-EP/opioid substance signaling pathway and the polarization of macrophages towards the M1 phenotype through NF-κB pathway. Regarding *microglia* and *neutrophils*, the existing data are primarily confined to chemotherapy-related complications such as chemotherapy- induced peripheral neuropathy (CIPN) and bone marrow suppression. There is still a dearth of direct evidence regarding the antitumor effect of acupuncture mediated by *mast cells*, necessitating further in-depth exploration. Acupuncture can prevent *neutropenia*, alleviate CIPN, and enhance the quality of life, thus serving as a synergistic treatment modality for tumors.

## 3 Modulation of adaptive immune cells

### 3.1 Helper T cells (*CD4^+^ T cells*)

*CD4^+^ T cells* differentiate into helper T (Th) cells and regulatory T cells (*Treg*s). Th cells include *Th1*, *Th2*, and *Th17* subsets, each with distinct cytokine profiles and functions. *Th1 cells* drive antitumor immunity by secreting proinflammatory cytokines, whereas *Th2 cells* promote tumor progression through immunosuppressive cytokines. The *Th2*-driven microenvironment facilitates immune evasion and metastasis (56, 57). Reciprocal cytokine suppression maintains the *Th1/Th2* balance; its disruption is a decisive factor in malignant tumor development (58). *Th17-*related cytokines enhance angiogenesis and immune evasion, promoting metastasis (59–61).

In a clinical trial of advanced gastric cancer patients post-second-line chemotherapy, acupuncture targeting ST36, LU10, and other acupoints improved immune parameters and quality of life. This dual modality restored *Th1/Th2* balance via upregulated T-box transcription factor (T-bet)/IFN-γ and suppressed GATA binding protein 3 (GATA3)/IL-4 mRNA in peripheral blood mononuclear cells (PB*MCs*), driving a *Th1*-polarized response. The immunomodulatory effect was further validated by decreased plasma IL-6, carbohydrate antigen 199 (CA199), and C-reactive protein (CRP), correlating with prolonged progression-free survival (PFS) and overall survival (OS) in the cohort (62).

In lung cancer patients post-thoracotomy, transcutaneous acupoint electrical stimulation (TAES) was applied at bilateral LI4 (Hegu), PC6 (Neiguan), SI3 (Houxi), and SJ6 (Waiguan) acupoints. Stimulation began 30 minutes prior to incision, followed by sessions at 20, 44, 68, 92, and 116 hours post-surgery. Cutaneous self-adhesive electrode pads (16 cm²) connected to a HANS-200 device delivered stimulation in standard dense-and-disperse mode for 30 minutes per session, alternating between 2 Hz and 100 Hz every 3 seconds (frequency: 2/100 Hz). The intensity was set at 4–12 mA to induce mild muscle contractions, with patients informed that current perception might vary. In the sham TAES group, identical electrical parameters were used, but stimulation was applied to sham points located 4.0 cm superolateral to the target acupoints (outside meridian pathways). Mechanistically, TAES modulated immune responses by reducing proinflammatory cytokines (IL-2, IFN-γ, IL-17) and enhancing anti-inflammatory IL-10. This shift promoted *Th1*/*Th17* balance while inhibiting *Treg*s, countering surgery-induced immunosuppression. Consequently, TAES-mediated immune homeostasis correlated with accelerated postoperative recovery (63).

Wang et al. treated Lewis lung cancer rat models with moxibustion and found that moxibustion enhanced the infiltration of *CD4^+^ T cells* and *Th1 cells* in the TME. Furthermore, the combination therapy of cisplatin and moxibustion significantly increased the proportions of *CD8^+^ T cells*, *CD4^+^ T cells*, *Th1* cells, *Th9 cells*, and *M1 Macrophages*. Additionally, the study demonstrated that both moxibustion and the combinatorial therapy reduced the expression of VEGF in tumor tissues. These findings suggest that the combination treatment strategy of cisplatin and moxibustion may offer a synergistic therapeutic approach for NSCLC, potentially improving clinical efficacy through enhanced immune modulation and TME regulation (29).

CIAA, an intervention implanting absorbable catgut into specific acupoints and an evolved form of traditional acupuncture and moxibustion, is a promising therapeutic approach for hepatocellular carcinoma (HCC). Liang et al. established an HCC rat model and performed CIAA treatment at ST36 and RN4 (Guanyuan) once every ten days for six sessions. This therapy effectively reduced mortality, alleviated HCC-related weight loss and improved mental status. The potential mechanism includes elevated *CD4^+^* and *CD8^+^ T cells* levels as well as decreased IL-10 expression. CIAA may relieve local immunosuppression in HCC by inhibiting the AKT pathway, thereby enhancing antitumor immunity and survival prognosis. Further investigation is necessary to optimize this treatment modality for potential clinical applications (64).

In a rat model of bone cancer pain (BCP) established by intratibial injection of Walker 256 mammary gland carcinoma cells, the study randomized rats into five groups: Control group, Sham BCP, BCP, BCP+EA, and BCP+Morphine. EA was applied bilaterally to ST36 and BL60 (Kunlun) using dilatational waves at 2/100 Hz frequency, with intensities escalating from 0.5 mA to 1 mA to 1.5 mA (10 min per intensity, 30 min total), administered every other day for 8 sessions. Morphine was given at 10 mg/kg via intraperitoneal injection on the same schedule. Results showed that while EA alleviated mechanical allodynia, its analgesic potency was weaker than that of morphine. Notably, EA significantly increased the proportions of splenic *CD3^+^CD4^+^* and *CD3^+^CD8^+^ T cells* subsets compared to morphine, indicating that moderate intensities (1-1.5 mA) optimize *T cells* activation. Concomitantly, EA elevated plasma IL-2 levels and its analgesic/immunomodulatory effects were partially reversed by naloxone, implicating an opioid-mediated mechanism. These findings demonstrate that EA effectively alleviates BCP-induced mechanical allodynia while enhancing cellular immunity, offering a non-pharmacological therapeutic approach for bone cancer pain (65).

In elderly patients undergoing gastrointestinal tumor resection, EA at GV20 (Baihui), LI4, PC6, HT7 (Shenmen), and bilateral ST36 upregulated *CD4*^+^ *T cells* and the *CD4^+^/CD8^+^* ratio at 24 hours postoperatively, while lowering TNF-α, IL-6, and IL-1β (which can cross the blood-brain barrier). EA at bilateral PC6 alone (2/100 Hz) also upregulated opioid receptors (μ-opioid receptor (MOR), δ-opioid receptor (DOR), κ-opioid receptor (KOR)) in the thymus and increased *CD4^+^* and *CD8^+^ T cells* populations, suggesting that peripheral opioid peptide activation contributes to EA’s immunomodulatory effects. Postoperative immunosuppression is closely linked to cancer metastasis risk; thus, EA may counteract immune dysregulation after cancer surgery (31, 66, 67).

### 3.2 Cytotoxic T cells (*CD8^+^ CTLs*)

*CD8^+^* cytotoxic T lymphocytes (*CTLs*) are the most critical effector cells for identifying and eliminating tumor cells. In the LLC model, moxibustion plus cisplatin increased *CD8^+^ T cells* infiltration into the TME (29). In HCC model, CIAA at ST36 and RN4 upregulated *CD8^+^ T cells* (64). In the BCP model, EA increased splenic *CD3^+^CD8^+^ T cells* subsets more effectively than morphine (65). In elderly gastrointestinal surgery, EA raised the *CD4^+^/CD8^+^* ratio and *CD8^+^ T cells* populations, with thymic opioid receptor upregulation suggesting enhanced *T cells* output from the central immune organ (66, 67).

In breast tumor-bearing mice, EA applied at the ST36 significantly reduced tumor volume and weight within 22 days post-implantation, accompanied by increased *CD8^+^ T cells* infiltration and cytotoxic activity. Additionally, EA downregulated proinflammatory cytokines, including IL-1β and TNF-α, at both local and systemic levels, thereby alleviating inflammatory infiltration within tumor tissues. Furthermore, the therapeutic effects of EA were attributed to the activation of the subdiaphragmatic vagus nerve, which suppressed systemic and local inflammatory cytokine production. These findings highlight the potential of EA as an adjunctive therapy for breast cancer by modulating immune responses and reducing inflammation (18).

In summary, acupuncture and its derivatives (EA, moxibustion, TAES, CIAA) regulate adaptive immunity by polarizing *helper T cell* subsets and enhancing *cytotoxic T cell* function. They also show potential in managing cancer-related pain and mitigating postoperative immunosuppression. As mechanisms become clearer, acupuncture-based therapies may complement conventional oncology care to provide more effective, personalized cancer treatment. (**Supplementary Figure 1**).

### 3.3 Regulatory T cells (Tregs)

*Regulatory T cells* (*Tregs*), a subset of *CD4^+^ T cells*, are fundamental to the regulation of immune homeostasis and tolerance. In TME, *Tregs* contribute to immunosuppression and tumor progression. *Treg*s are known to heavily infiltrate tumor tissues and are strongly associated with poor prognosis in cancer. *Tregs* exert immunosuppressive effects mainly via the secretion of inhibitory cytokines, including IL-10, IL-4, and TGF-β1. Conversely, elevated levels of interferon-gamma (IFN-γ) have been shown to inhibit the formation and function of *Treg*s (68, 69).

In a sarcoma transplantation model, moxibustion decreased the serum levels of IL-4, IL-10, and TGF-β1, while increasing the level of IFN-γ in TME. The antitumor efficacy of moxibustion against sarcoma might be ascribed to the reduction of circulating *Treg* counts and the modulation of *Treg* infiltration within the TME. Moxibustion intervention inhibits *Treg*-mediated immunosuppression and enhances antitumor immune responses. Overall, moxibustion functions as an effective intervention for soft tissue sarcomas by reducing *Treg* infiltration in the peripheral blood and TME, thus constraining tumor immune escape and augmenting antitumor immunity (70).

Acupuncture can inhibit the excessive activity of *Tregs* in the tumor microenvironment and play a role in suppressing tumor growth. However, for malignancies with high incidence and mortality (including breast cancer, lung cancer, colorectal cancer, *etc.*), specific data regarding whether acupuncture can suppress tumor immune escape remain lacking. Exploring the mechanism of acupuncture in regulating and improving the symptoms of inflammatory bowel disease and cerebrovascular disease can provide valuable therapeutic strategies for cancer-related diseases (**Supplementary Figure 2**; **Table 3**).

**Table 3:** Acupuncture and moxibustion therapy in anti-cancer immune effects. ↑ increased / upregulated; ↓ decreased / downregulated.

Cell type	Therapy	Acupoints and dosage	Immune efficacy	Mechanism/Pathway	Cancer type	Reference
*CD4*^+^ T and *CD8*^+^ T cells	Acupunctur and moxibustion	Acupuncture: ST36, LU10, SP18, EX-B4, GB42, 30min Moxibustion: ST16, RN12, ST36, BL21, BL23, BL43 (3 cones/point).	↑Th1, T-bet and IFN-γ (P<0.001) ↓Th2, GATA3 and IL-4, IL-6 (P<0.01)	Suppressing IL-6/CRP pathways	Gastric cancer	(62)
	Transcutaneous acupoint electrical stimulation (TAES)	LI4, PC6, SI3 and SJ6 (2/100 Hz, 4–12 mA, 30 min)	↑Th1, Th17, IL-2, IFN-γ, and IL-17 T-bet, RORγ t (P< 0.05) ↓Th2, IL-10 GATA3 (P< 0.05)	Regulation of Th1/Th2/Th17/Treg balance, potential vagal and opioid-mediated modulation	Lung cancer	(63)
	Moxibustion	ST36 (15 min, 5 times/ Week, 2weeks)	↑*CD8*^+^ CTLs, *CD4*^+^ T cells, Th1, Th9	Improve vascularnormalization, suppress VEGF expression	NSCLC	(29)
	Catgut implantation at acupoints (CIAA)	ST36, RN4 (Every 10 days for 6 sessions)	↑*CD4*^+^ and *CD8*^+^ T cells (P< 0.05) ↓IL-10 (P<0.0005)	Suppress AKT pathway	HCC	(64)
	EA	ST36, BL60 (2Hz, 1.0mA, 30min, 7days)	↑IL-2 (P<0.01), *CD4*^+^ and *CD8*^+^ T cells	Opioid-mediated pathway	Bone cancer pain, BC	(65)
	EA	GV20, LI4, PC6, HT7, ST36 (20 min pre-anesthesia induction)	↑*CD4*^+^ T cells; ↓TNF-α, IL-6, IL-1β (P < 0.05).	Bidirectional regulation of HPA	Gastrointestinal tumor resection	(67)
	EA	ST36 (2/15 Hz,0.1 mA,30 minutes, QOD)	↑*CD8*^+^ T cells, perforin (P<0.05), granzyme B​ (P< 0.01) ↓IL-1β, TNF-α	Activate subdiaphragmatic vagus nerve (c-Fos↑)	BC	(18)

## 4 Modulation of non-immune stromal cells

Within the TME, in addition to tumor cells and immune cells, non-immune stromal cells (including vascular endothelial cells (VECs), and cancer-associated fibroblasts (CAFs)), play crucial roles in tumor progression and therapy response (71). Currently, studies investigating the regulation of non-immune cells within the TME by acupuncture have predominantly focused on *ECs*. *ECs* are critical for tumor growth beyond microscopic size through angiogenesis, with VEGF as a master regulator, and they facilitate metastasis via bidirectional tumor-vessel interactions, such as enabling tumor cells intravasation/extravasation and modulating the premetastatic niche. Additionally, tumor-associated *ECs* interact with the immune system, influencing *T cells* infiltration and responses to immunotherapy, while anti-angiogenic therapies normalize tumor vasculature to improve treatment efficacy (72, 73) (**Supplementary Figure 3**).

In tumor-bearing mice, EA applied in the up, down, left, and right directions relative to the tumor exerts regulatory effects on angiogenesis and stromal remodeling. Its primary mechanism involves suppressing glyoxalase 1 (GLO1) within *ECs*, which in turn mitigates glycolysis and angiogenesis, thereby promoting vascular normalization. It notably reduces GLO1 expression in endothelial cells of 4T1 xenografts, and analogous to GLO1 knockdown, inhibits angiogenesis, as demonstrated in the *in vivo* matrigel plug angiogenesis assay. *In vitro*, both pharmacological inhibition and genetic silencing of GLO1 in human umbilical vein endothelial cells (HUV*ECs*) impede their proliferation, migration, tube formation, and sprouting, while promoting apoptosis, which is linked to the downregulation of the methylglyoxal-glycolytic pathway. Furthermore, EA enhances endothelial cell adhesion, ameliorates the integrity of the vascular basement membrane, and increases pericyte coverage, all of which contribute to the improvement of vascular structure and function. It also downregulates the expression of angiogenic factors such as VEGFA and PDGFB in endothelial cells. Therefore, peri-tumoral EA promotes vascular normalization in 4T1 breast cancer xenografts. When combined with paclitaxel chemotherapy, peri-tumoral EA enhances the chemotherapeutic effect. Endothelial cell GLO1 serves as a key target through which peri-tumoral EA regulates angiogenesis (74).

In contrast to the immune regulation discussed in previous chapters, acupuncture can directly act on *ECs* to promote vascular normalization and improve drug delivery. Vascular normalization effectively alleviates tumor hypoxia, a key driver of immunosuppression characterized by the accumulation of *Tregs* and *MDSCs*. Thus, the vascular normalization induced by acupuncture may indirectly potentiate antitumor immunity, offering a novel perspective for understanding the multi-target integrative effects of acupuncture.

## 5 Discussion

### 5.1 Acupuncture-mediated TME remodeling and the core therapeutic principle of “Fuzheng”in TCM

In traditional Chinese medicine, the pathogenesis of tumors is ascribed to a deficiency of Zhengqi (the healthy qi) and an excess of Xieqi (the accumulation of pathogenic factors), and the core therapeutic approach is Fuzheng Quxie (supporting the healthy qi and eliminating the pathogenic factors). The modern immunological foundation of this theory is closely related to the remodeling of TME. As presented in this review, acupuncture and moxibustion comprehensively reshape the immunosuppressive TME via multiple pathways, which can be considered a specific manifestation of the Fuzheng principle.

On one hand, acupuncture enhances antitumor immune surveillance by regulating innate and adaptive immune cells: it activates *NK cells* and *macrophages*, promotes *Th1* polarization, and enhances *CD8^+^ CTLs* cytotoxicity. On the other hand, acupuncture suppresses immunosuppressive cells including *Tregs* and *M2* macrophages, thereby restoring the balance between antitumor and pro-tumor immune networks.

### 5.2 Differential effects of acupuncture and moxibustion modalities

According to the evidence summarized in this review, MA, EA, and moxibustion exert distinct regulatory profiles and are suitable for different clinical scenarios.

MA, a form of mechanical stimulation of acupoints, exerts bidirectional immune regulation suitable for patients requiring gentle modulation.

EA provides controlled electrical stimulation with precise parameters, making it ideal for standardized clinical protocols (9), Notably, EA exhibits the most potent TME remodeling capacity. EA at ST36 or peritumoral sites promotes M1−type TAM polarization, activates the STING signaling pathway, enhances *NK cells* and *CD8^+^ T cells* cytotoxicity, and achieves vascular normalization. EA is therefore suitable for combination with chemotherapy or anti−PD−1 immunotherapy, especially in MSS−CRC and breast cancer models, to enhance antitumor efficacy.

Moxibustion exerts its effects through the synergistic action of thermal effects, radiation effects, and pharmacological effects of mugwort. Its near-infrared radiation penetrates subcutaneous tissues, while the active components of mugwort contribute to its therapeutic actions (75, 76). Moxibustion offers thermal stimulation that may be particularly effective for patients with yang deficiency patterns. Moxibustion shows prominent superiority in alleviating chemotherapy-related myelosuppression: it elevates white blood cells and absolute *neutrophil* counts, reduces the risk of severe neutropenia and febrile neutropenia, inhibits β−adrenergic signaling, promotes *DCs* maturation, and improves patient quality of life. Moxibustion is therefore recommended as a routine adjuvant intervention during chemotherapy cycles to maintain hematopoietic and immune function.

### 5.3 Mechanical stimulation of acupuncture in immune regulation

Mechanical stimulation induced by acupuncture (e.g., lifting, thrusting, and rotation) exerts immunomodulatory effects that are contingent upon intact peripheral neural circuits, including the vagus and sciatic nerves. Any pathological conditions that impede these pathways, such as tumor burden and associated neuropathy, attenuate acupuncture-mediated modulation of central immunity. Mechanistic research indicates that peripheral acupuncture stimulation enables the vagus nerve to function as a core efferent arm of the cholinergic anti-inflammatory pathway, suppressing proinflammatory cytokine release through the activation of macrophage α7 nicotinic acetylcholine receptors. Moreover, electrical stimulation of the sciatic nerve activates a distinct sciatic-vagus reflex arc, which ultimately triggers the release of adrenal medullary dopamine and elicits systemic anti-inflammatory responses via D1 receptor signaling. Vagotomy completely abolishes these effects, validating the essential role of vagal transmission. The autonomic nervous system, composed of sympathetic and parasympathetic divisions, serves as a fundamental element of acupuncture-evoked neuroimmunomodulation, through which acupuncture exerts anti-inflammatory effects. Notably, any conditions leading to structural or functional neural damage, such as chemotherapy, radiotherapy, or tumor infiltration, may undermine the therapeutic potential of acupuncture. Future studies should systematically assess the influence of neuropathy on clinical outcomes in cancer and related populations (77–79).

### 5.4 Effects of stimulation parameters on immune regulation and implications for clinical standardization

A key pattern revealed in this review is that the immune effects of acupuncture are parameter−dependent.

For EA, parameter optimization determines immune activation thresholds. In MSS-CRC, EA at ST36 with 1.0 mA/10 Hz yields optimal STING−dependent TME remodeling, which is superior to 0.5 mA and 1.5 mA. Low−frequency EA (2 Hz) mainly elevates *NK cells* proportions and alleviates CIPN, whereas dense-sparse wave (3-4 Hz/15-20 Hz) more effectively promotes vascular normalization and TAM repolarization. For grain−sized moxibustion, 3 cones and 7 cones at ST36 produce comparable *NK cells* activation, indicating a saturation effect rather than dose dependency. These findings highlight the necessity of standardized stimulation protocols in clinical practice. To achieve stable antitumor immune effects, clinical applications should specify acupoint combinations (e.g., ST36 as the core acupoint), stimulation modality (EA for TME remodeling, moxibustion for myelosuppression), intensity, frequency, wave form, and duration rather than using empirical or inconsistent parameters.

## 6 Conclusion

In summary, acupuncture and moxibustion have proven valuable as adjunctive oncological therapies, alleviating a wide spectrum of cancer-associated symptoms and treatment-related toxicities (80). Acupuncture not only suppresses tumor growth but also, when combined with pharmacologic agents, reduces adverse effects while promoting vascular normalization and alleviating hypoxia. Moxibustion, in parallel, mitigates chemoradiotherapy side effects and bolsters immune function. Together, these actions provide a novel therapeutic rationale for integrating TCM syndrome differentiation with immunotherapy.

Nevertheless, current mechanistic evidence regarding acupuncture-mediated immune modulation and TME remodeling remains largely preclinical. Although numerous clinical studies have focused on improving cancer-related symptoms, rigorous clinical investigations directly verifying the intratumoral immunomodulatory effects of acupuncture are limited. Data on intratumoral *Treg* modulation are particularly lacking. Biopsy-driven, well-controlled clinical trials are needed to validate efficacy and inform personalized acupuncture regimens. Genomic sequencing and bioinformatics could further dissect how acupuncture regulates *Tregs* at the molecular level, paving the way for more effective acupuncture-based interventions-standalone or combinatorial-to enhance antitumor immunity and patient outcomes.

Future research should focus on integrating acupuncture into precision cancer treatment. For instance, quantifying immune cells and cytokines in patient biopsies or peripheral blood could guide the selection of acupoints, stimulation parameters, and treatment schedules tailored to individual patients, thereby optimizing efficacy, curbing toxicity, and improving prognosis.
