# Supplementary figures and images for: Antitumor effects of acupuncture and moxibustion: from immune modulation to tumor microenvironment remodeling
Source: Front Immunol. 2026 Jun 18;17:1772199. doi: 10.3389/fimmu.2026.1772199 (PMC13323237; doi:10.3389/fimmu.2026.1772199)

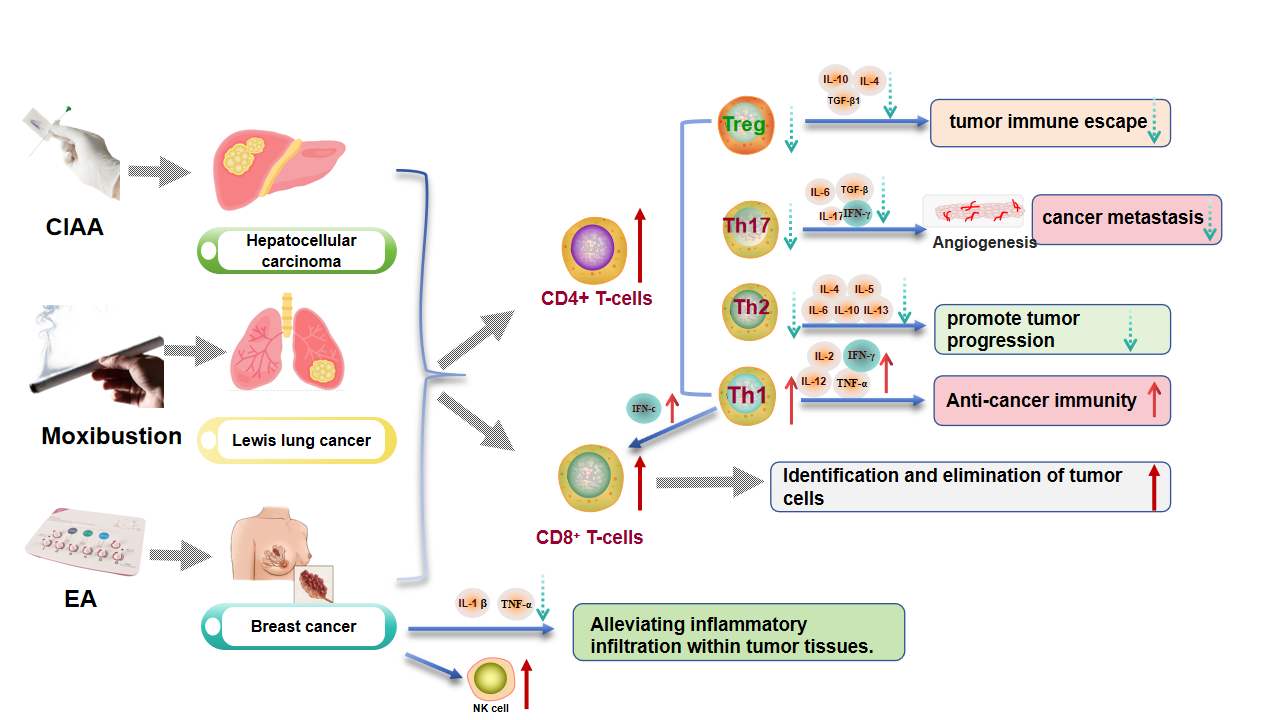

Supplement: Supplementary file 1 [file Image1.tif]

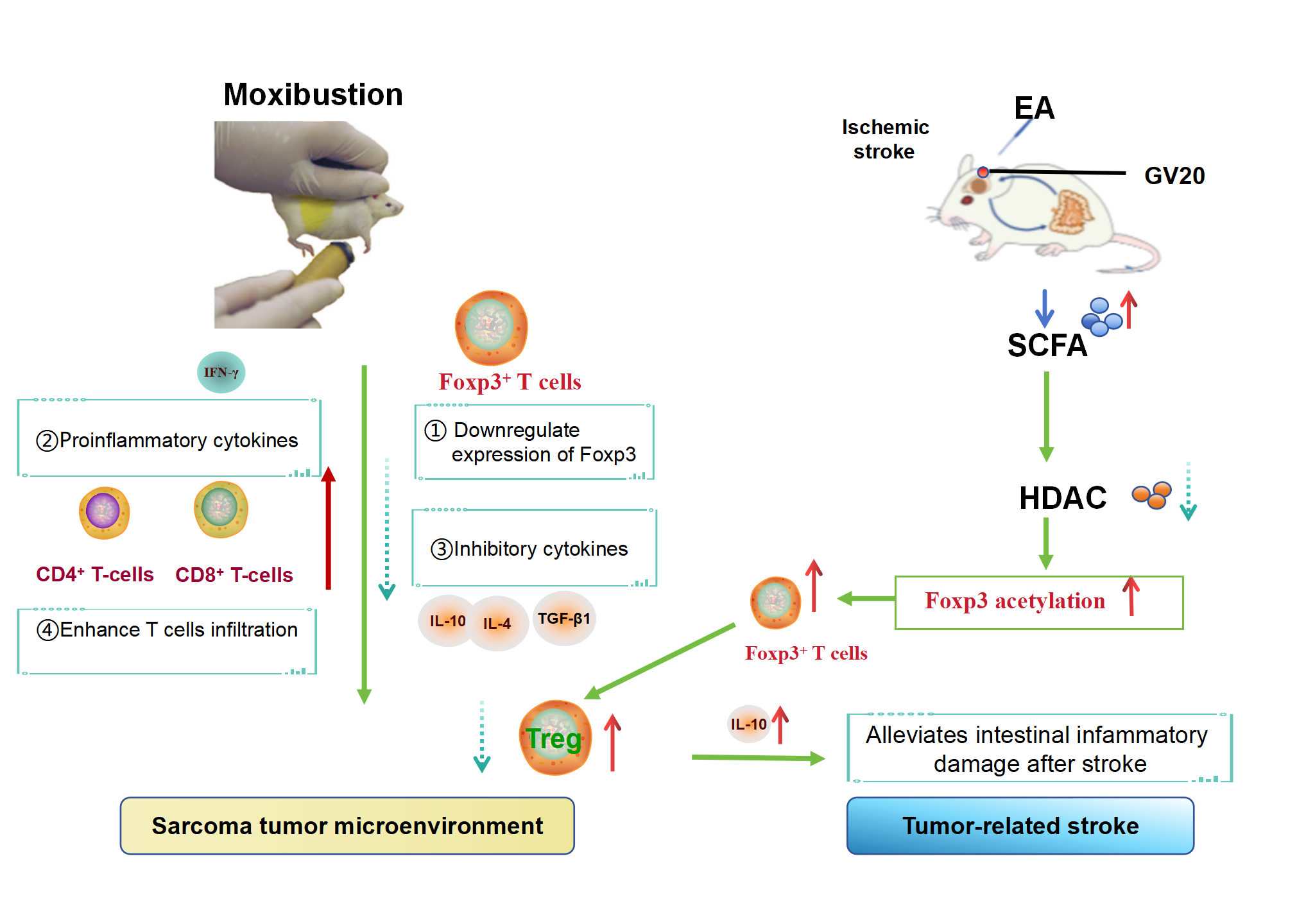

Supplement: Supplementary file 2 [file Image2.tif]

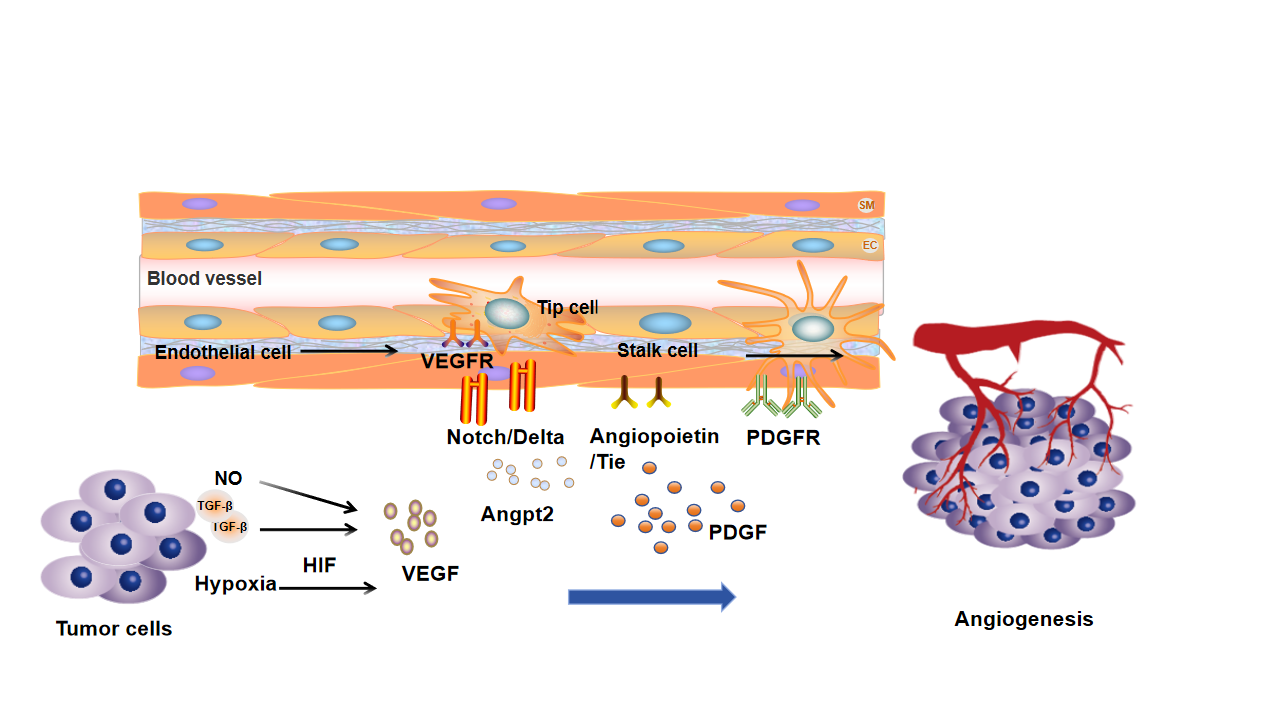

Supplement: Supplementary file 3 [file Image3.tif]
